# p27 Expression in Wild‐Type KRAS Colon Cancer

**DOI:** 10.1111/jcmm.71248

**Published:** 2026-06-22

**Authors:** Sonja Marinović, Iva Paladin, Anita Škrtić, Tina Catela Ivković, Donatella Verbanac, Sanja Kapitanović

**Affiliations:** ^1^ Division of Molecular Medicine Ruđer Bosković Institute Zagreb Croatia; ^2^ TEVA Group Member R&D, PLIVA Croatia Ltd Zagreb Croatia; ^3^ Department of Pathology Clinical Hospital Merkur Zagreb Croatia; ^4^ Center for Translational Genomics Faculty of Medicine Lund Sweden; ^5^ Department of Medical Biochemistry and Haematology University of Zagreb, Faculty of Pharmacy and Biochemistry Zagreb Croatia

**Keywords:** colon adenocarcinoma, KRAS, *miR‐221/222*, p27, *V109G* SNP

## Abstract

p27, a cyclin‐dependent kinase inhibitor, functions as a tumour suppressor in the nucleus but may acquire oncogenic properties when mislocalized to the cytoplasm. While KRAS mutations can induce p27 phosphorylation and cytoplasmic retention, the regulation and significance of p27 expression in wild‐type (WT) KRAS colorectal cancer (CRC) remain unclear. This study investigated the relationship between WT KRAS status and p27 localization, as well as the potential roles of miR‐221/222 expression and the CDKN1B V109G polymorphism in CRC susceptibility. Immunohistochemical analysis of 50 WT KRAS CRCs and adjacent normal tissues revealed the highest percentage of p27‐positive cells in the superficial layer of normal mucosa and significantly fewer in the tumour center. WT KRAS tumours with KRAS expression showed increased p27 expression and predominant cytoplasmic localization at the invasive front, suggesting altered p27 subcellular distribution. *miR‐221/222* expression showed no correlation with p27 levels, and the CDKN1B V109G polymorphism was not associated with CRC risk. This study is the first to examine p27 localization in WT KRAS CRC. The observed association between WT KRAS expression and cytoplasmic p27 localization highlights a potential mechanism contributing to tumour progression through altered p27 function.

## 1 Introduction

Colorectal cancer (CRC) is one of the most common cancers worldwide and the second leading cause of cancer‐related death globally. It often starts as a small, benign adenoma that can progress to malignancy over time due to genetic alterations driving the transformation of normal colonic epithelium into carcinoma [1]. Mutations in *Kirsten rat sarcoma virus oncogene homologue* (*KRAS*), particularly at codons 12 and 13, are among the most common oncogenic drivers in human cancers, including colorectal cancer [2]. Mutant KRAS protein leads to constitutive activation of downstream signalling pathways, promoting cell proliferation and survival [3] and contributing to more aggressive tumours resistant to therapy [4, 5]. KRAS plays a role in regulating the cell cycle, which is tightly modulated by cyclins, cyclin‐dependent kinases (CDKs) and CDK inhibitors. Several studies suggested that intracellular pathways downstream of KRAS such as mitogen‐activated protein kinase (MAPK) and phosphoinositide 3‐kinase (PI3K) can promote phosphorylation of CDK inhibitor p27^Kip1^ (p27), leading to its ubiquitination and subsequent proteasomal degradation or cytoplasmic mislocalization [1, 6].

p27 belongs to the CDK interacting protein/Kinase inhibitory protein (CIP/KIP) family of CDK inhibitors and functions as a haploinsufficient tumour suppressor, with both CDK‐dependent and CDK‐independent roles. It functions primarily by inhibiting cyclin/cyclin‐dependent kinase complex activity, particularly CDK2, which is crucial for cell cycle progression [7]. Loss of p27 function allows unchecked cell cycle progression contributing to the uncontrolled proliferation characteristic of cancer cells [8]. In human cancers, mutations or deletions in the *CDKN1B* gene, which encodes p27, are rare [9], however, a single nucleotide polymorphism of *CDKN1B* gene p.V109G (rs2066827) has been associated with reduced transcription rates, decreased p27 protein levels and correlated with cancer incidence and prognosis [10, 11, 12].

Several studies have demonstrated that p27 expression is decreased in late‐stage colon cancers compared to early‐stage tumours, indicating that loss of p27 may be a progressive event in colon carcinogenesis that could drive tumour progression and metastasis formation [13, 14]. Loss of p27 protein function can occur through various mechanisms including increased protein degradation, mislocalization or transcriptional downregulation [15, 16]. In normal cells, p27 is predominantly localized in the nucleus, where it binds to Cyclin E/CDK2, blocking the kinase activity and preventing transition from the G1 to the S phase. Conversely, cytoplasmic p27 regulates vesicle trafficking, cytokinesis, cytoskeletal dynamics, motility and invasiveness. Due to p27's vital roles in the nucleus and cytoplasm, cells strictly regulate its levels and location, as disruptions can lead to uncontrolled proliferation and increased risk of tumour formation and progression [17, 18, 19].

miRNA induced downregulation of cyclin‐CDK inhibitors is thought to be an important mechanism in human carcinogenesis [20, 21, 22]. In the context of p27, *miR‐221‐3p* and *miR‐222‐3p* share a conserved seed region capable of binding to the 3′ untranslated region (UTR) of *CDKN1B* mRNA leading to its degradation or translational repression, thereby promoting increased cell proliferation [23]. In addition, these miRNAs are frequently upregulated in tumours and are associated with poor prognosis [24, 25, 26]. Moreover, several studies have shown that *miR‐221*/*222* are overexpressed in mutated KRAS cancer cell lines [6, 27, 28], however, much less is known about their expression patterns in tumours harbouring wild‐type (WT) KRAS. Unlike mutated KRAS protein, which is constitutively active and drives tumour progression, WT KRAS retains its responsiveness to upstream regulatory signals, allowing for more controlled cellular behaviour [2].

Chemotherapy, with or without the addition of anti‐EGFR therapy, remains the standard of care for patients with WT KRAS colorectal cancer, who currently have limited therapeutic options. Therefore, the identification of novel clinically relevant biomarkers and therapeutic targets remains an urgent priority for this patient population. In WT KRAS tumours, p27 misregulation may compensate to sustain proliferation and survival, helping drive tumour progression in the absence of constitutively active KRAS. This makes p27 a potentially actionable target or marker in tumours without KRAS mutations. Therefore, this study aimed to investigate the differences in the expression of CDK inhibitor p27 and its regulation in patients with wild‐type KRAS colorectal cancer.

## 2 Materials and Methods

### 2.1 Patients

A total of 330 colorectal cancer samples were collected, of which 130 met the molecular inclusion criteria, defined as either isolated KRAS mutations (codons 12/13) or wild‐type KRAS status. The remaining 200 samples were excluded due to additional alterations (mutations in *TP53, PIK3CA, BRAF, CTNNB1*) or molecular instability (MSI, EMAST) [29]. Adequate RNA quality for mRNA/miRNA analysis was obtained in 110 of the 130 selected cases (43 mutant KRAS, 67 WT KRAS). Tumour and adjacent normal colon tissue samples, located approximately 15 cm from the tumour edge, were collected at Clinical Hospital Merkur during surgery using standard clinical procedures. None of the patients received preoperative radiotherapy or chemotherapy. The collected tissue samples were immediately frozen in liquid nitrogen and stored at −80°C for subsequent DNA and RNA extraction. Additionally, tumour and adjacent normal mucosa samples were preserved via formalin fixation and paraffin embedding. Routine haematoxylin and eosin staining was performed for histopathological evaluation. Clinical data, including age, sex, tumour size, differentiation grade, Dukes' stage and tumour location, were recorded for each patient.

The study was approved by the Ethics Committee of Clinical Hospital Merkur, Zagreb (May 24, 2016, UR. BR. 03/1‐4723) and conducted in accordance with the ethical principles of the Declaration of Helsinki. Written informed consent was obtained from all participants.

### 2.2 KRAS Mutation and Protein Expression

*KRAS* mutations at codons 12 and 13 were identified using PCR‐RFLP (polymerase chain reaction–restriction fragment length polymorphism), and KRAS protein expression was assessed via RT‐PCR and immunohistochemistry, as previously published [30]. As previously shown, mutated KRAS tumours consistently exhibited high protein expression by immunohistochemistry. In contrast, within the WT KRAS group, approximately 60% of samples were positive for KRAS protein expression, while the remaining 40% showed no detectable KRAS expression [30].

### 2.3 mRNA And miRNA Expression Analysis

Total RNA was isolated from 110 samples of snap‐frozen colon adenocarcinoma tissues and matched adjacent normal tissues using Trizol reagent, following the manufacturer's guidelines. After assessing RNA quantity and quality, the samples were used for mRNA and miRNA expression analyses. cDNA synthesis was carried out using the High‐Capacity cDNA Reverse Transcription Kit (Applied Biosystems, Thermo Fisher Scientific, Waltham, MA, USA) as per the provided protocol. For mRNA synthesis, random hexamer primers were used, whereas specific primers were used for *miR‐221, miR‐222* and the reference gene *RNU48*.

*CDKN1B* (p27) mRNA expression was quantified using the TaqMan Gene Expression Assay (Hs01096154_m1, Applied Biosystems, Thermo Fisher Scientific, Waltham, MA, USA), with *ACTB* (Hs01060665_g1, Applied Biosystems, Thermo Fisher Scientific, Waltham, MA, USA) serving as the internal control. Expression levels of *miR‐221‐3p and miR‐222‐3p* were assessed using TaqMan MicroRNA Assays (Applied Biosystems, Thermo Fisher Scientific, Waltham, MA, USA; Assay IDs: 000524 and 000525) and normalized to *RNU48* (Assay ID: 001006, Applied Biosystems, Thermo Fisher Scientific, Waltham, MA, USA). Relative expression levels of both mRNA and miRNA were calculated using the comparative Ct (ΔCt) method [31].

### 2.4 Immunohistochemistry

From the total of 67 WT KRAS samples for which mRNA data were obtained, we were able to obtain 50 formalin‐fixed paraffin‐embedded (FFPE) blocks for further analysis. The remaining 17 WT KRAS were excluded solely on technical ground since the corresponding FFPE tissue blocks did not permit reliable sectioning. Sections of sporadic CRC samples were deparaffinized in xylene, rehydrated through a graded ethanol series and rinsed in phosphate‐buffered saline (PBS). Antigen retrieval was performed by microwave heating in citrate buffer (10 mM citric acid, 0.05% Tween 20, pH 6.0). Non‐specific binding was minimized by applying DAKO Protein Block Serum‐Free (DAKO, Denmark) in a humidified chamber for 10 min at room temperature. After removing excess blocking solution, slides were incubated overnight with a primary mouse monoclonal antibody against p27 (AB_397636, BD Transduction Laboratories, Franklin Lakes, NJ, USA). Negative controls were prepared by excluding the primary antibody. The slides were then washed three times with PBS. Endogenous peroxidase activity was blocked by incubating the slides in PBS containing 3% hydrogen peroxide (Sigma‐Aldrich, Germany) for 10 min in the dark. For visualization, the DAKO EnVision + System, HRP (DAB) (DAKO, Denmark) was used following the manufacturer's protocol. Slides were counterstained with haematoxylin, dehydrated and mounted with Entellan (Sigma‐Aldrich, Germany). The extent of nuclear and cytoplasmic p27 expression was visually assessed only on epithelial cells, specifically on normal mucosal epithelial cells (assessed in both the superficial luminal epithelium (N_sup_) and the cryptal compartment (N_cry_)) and on neoplastic epithelial/tumour cells (assessed separately in the tumour center (T1) and at the invasive tumour front (T2)). Stromal cells, including fibroblasts, endothelial cells and inflammatory/immune cells, were systematically excluded throughout the study from both p27 positivity and staining intensity assessments. p27 immunoreactivity was evaluated semi‐quantitatively on the same histological slides in both tumour and normal tissues, enabling direct intra‐sample comparison of staining patterns under identical experimental conditions. For each compartment, p27 expression was assessed according to both the percentage of positive cells that quantifies the spatial extent of p27 expression as well as the staining intensity, graded as 0 (absent), 1 (weak), 2 (moderate), or 3 (strong) which corresponds to the degree of translational output or protein stability in the expressing cell population.

### 2.5 SNP Genotyping

The single nucleotide polymorphism (SNP) *V109G* (rs2066827), located in exon 2 of the *CDKN1B* gene, was genotyped using predeveloped TaqMan allelic discrimination assays and TaqPath ProAmp Master Mix (Applied Biosystems, Thermo Fisher Scientific, Waltham, MA, USA), following the manufacturer's protocol. Genotyping was performed via real‐time PCR using an ABI PRISM 7300 sequence detection system (Applied Biosystems, Foster City, CA, USA). Each experiment included positive controls representing all three possible genotypes, as well as a no‐template control run alongside the test samples for accuracy and validation.

### 2.6 Statistical Analysis

Statistical analyses were performed using the GraphPad Prism software (GraphPad Software, San Diego, CA, USA). The normality of continuous data was assessed prior to statistical testing using both the Shapiro–Wilk and Kolmogorov–Smirnov tests [32]. Differences in mRNA and miRNA expression between tumour and adjacent normal tissues were evaluated using the Wilcoxon signed‐rank test. Comparisons among three or more groups were conducted using the Kruskal–Wallis test followed by Dunn's post hoc test. Fisher's exact test was applied to contingency tables to analyse the difference in p27 immunohistochemical expression intensity and subcellular localization. For the correlation between the percentage of p27 positive cells and either *miR‐221‐3p* or *miR‐222‐3p* in tumour tissues, Spearman's rank test was used. The genetic association of the *V109G* SNP was evaluated by calculating pooled odds ratios (ORs) with corresponding 95% confidence intervals (CIs). Hardy–Weinberg equilibrium (HWE) was evaluated using a chi‐square goodness‐of‐fit test (*p* < 0.05). Data are presented as means ± standard error of the mean (SEM) or as box‐and‐whisker plots (5th to 95th percentiles). Statistical significance thresholds were set at *p < 0.05, **p < 0.01, ***p < 0.001, ****p < 0.0001.

## 3 Results

### 3.1 *CDKN1B* Expression Does Not Correlate With KRAS Mutation Status in CRC

**FIGURE 1:**
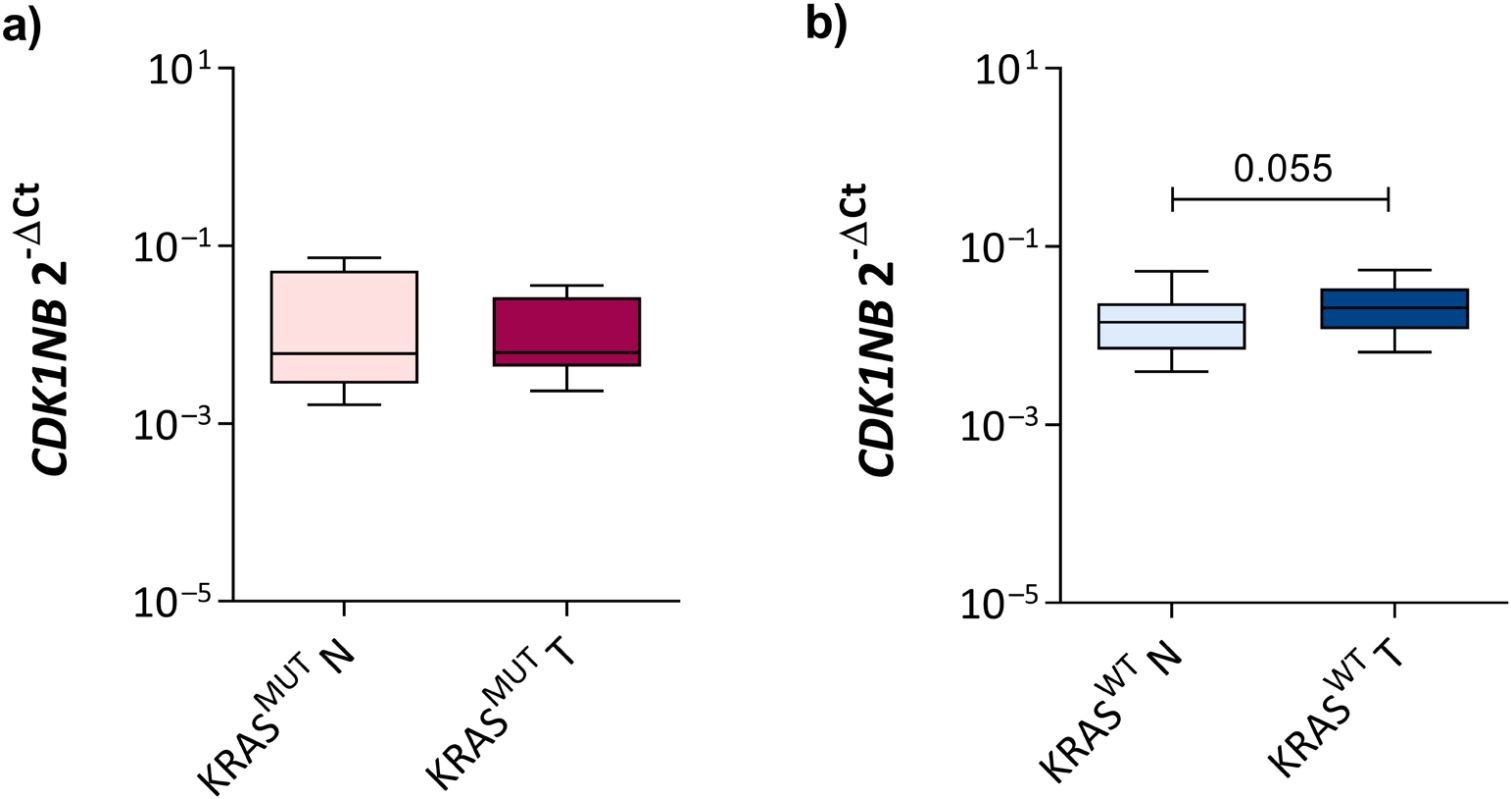
Analysis of CDKN1B (p27) gene expression in CRC. CDKN1B expression in tumour‐adjacent normal tissue (N) and tumour tissue (T) from patients with (a) mutated KRAS (KRAS^MUT^) or (b) wild‐type KRAS (KRAS^WT^) CRC. Figure 1a,b are presented as box‐and‐whisker plots (5th–95th percentiles) and were analysed using the Wilcoxon signed‐rank test.

Since several studies have proposed a relationship between KRAS and p27 expression, we assessed *CDKN1B* mRNA expression in WT KRAS and mutated KRAS tumour samples. Our analysis revealed no significant difference in *CDKN1B* expression between mutated KRAS tumour and adjacent normal tissue (*p* = 0.391) (Figure 1a), while WT KRAS tumours exhibited a modest, non‐significant increase in *CDKN1B* mRNA levels compared to adjacent normal tissue (*p* = 0.055) (Figure 1b). These results suggest that KRAS mutation status does not influence *CDKN1B* mRNA expression in colorectal cancer.

### 3.2 p27 Expression Depends on KRAS Protein Expression in WT KRAS CRC

Given the trend towards increased *CDKN1B* expression in WT KRAS tumours, we next investigated whether this was reflected at the protein level and whether WT KRAS protein expression has any correlation with p27 expression or subcellular localization.

**FIGURE 2:**
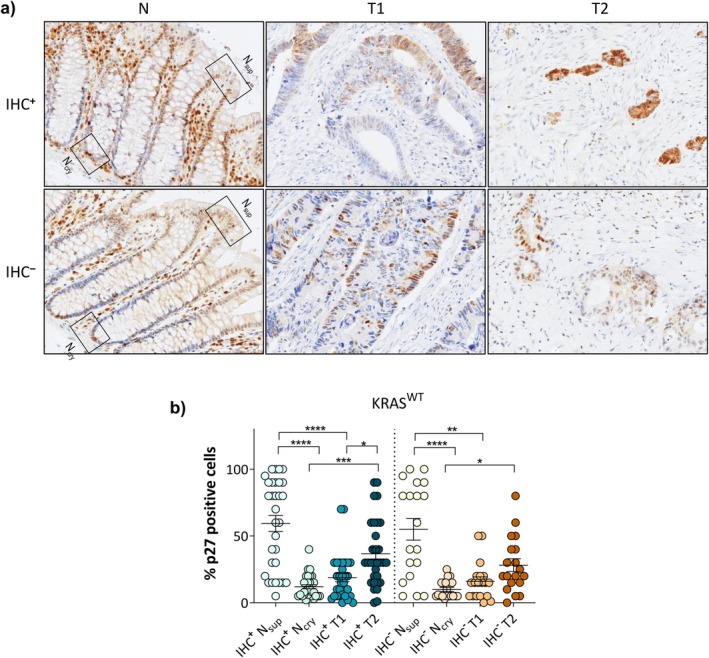
Immunohistochemical expression of p27 in wild‐type KRAS colorectal cancer. (a) Representative images of p27 protein expression in adjacent normal tissue (N), tumour center (T1) and invasive tumour front (T2) from tumour samples with (IHC^+^) or without (IHC^−^) detectable KRAS protein expression. (b) Percentage of p27‐positive cells in superficial layer (N_sup_) or base of Lieberkühn crypts (N_cry_) of adjacent normal tissue, tumour center (T1) and invasive tumour front (T2) in samples stratified by KRAS protein expression (IHC^+^ or IHC^−^). N, T1 and T2 regions were all collected from the same individual (magnification, ×400). Data are presented as means ± SEM and were analysed by Kruskal‐Wallis test followed by Dunn's correction (p* < 0.05, p** < 0.01, p*** < 0.001, p**** < 0.0001).

A total of 50 sporadic WT KRAS CRC samples, comprising 31 with immunohistochemically detectable KRAS protein expression (IHC^+^) and 19 without KRAS protein presence (IHC^**−**^), were assessed for p27 protein expression. For the analysis, four tissue regions were evaluated by immunohistochemistry: superficial layer with terminally differentiated cells (N_sup_) and the base of Lieberkühn crypts (N_cry_) in adjacent normal mucosa, then the tumour center (T1) and the invasive tumour front (T2) (Figure 2a). Regardless of KRAS protein presence, in both groups, a significantly higher percentage of p27‐positive tumour cells was observed in N_sup_ compared to N_cry_ (*p* < 0.0001 for both groups; Figure 2b) and T1 (*p* < 0.0001 for IHC^+^ group; *p* = 0.002 for IHC^**−**^ group; Figure 2b). Furthermore, T2 samples showed a significantly greater proportion of p27‐positive cells relative to N_cry_ (*p* = 0.0001 for IHC^+^ and *p* = 0.026 for IHC^**−**^; Figure 2b). The only difference between T1 and T2 was observed in the samples positive for KRAS expression (IHC^+^), where T2 exhibited a higher percentage of p27‐positive cells compared to T1 (*p* = 0.021; Figure 2b).

Overall, p27 immunoreactivity in all WT KRAS samples was most pronounced in the base of Lieberkühn crypts (N_cry_), showing significantly higher staining intensity compared to the superficial layer of adjacent normal mucosa (N_sup_) (*p* = 0.0002 for IHC^+^ group and *p* = 0.020 for IHC^**−**^ group) and T1 (*p* < 0.0001 for IHC^+^ group; *p* = 0.001 for IHC^**−**^ group) (Table 1). However, in WT KRAS IHC‐positive samples we also observed difference between N_cry_ and T2 (*p* = 0.022) and a trend towards difference in staining intensity between the T1 and the T2; however, this did not reach statistical significance (*p* = 0.050; Table 1).

**TABLE 1:** P27 IHC expression intensity in normal (N_sup_ and N_cry_), tumour center and invasive tumour front tissues stratified by WT KRAS protein expression.

27 score	0	1	2	3	*p*	
KRAS IHC+						
Nsup	0 (0)	20 (64.5)	11 (35.5)	0 (0)	**0.0002** ^a^***	**< 0.0001** ^d^****
Ncry	0 (0)	4 (12.9)	27 (87.1)	0 (0)	0.369^b^	**0.022** ^e^*
T1	2 (6.5)	22 (71.0)	7 (22.5)	0 (0)	0.220^c^	0.050^f^
T2	1 (3.2)	13 (42.0)	16 (51.6)	1 (3.2)		
KRAS IHC^**−**^						
N_sup_	0 (0)	11 (57.9)	8 (42.1)	0 (0)	**0.020** ^a^*	**0.001** ^d^**
N_cry_	0 (0)	4 (21.0)	15 (79.0)	0 (0)	0.200^b^	0.109^e^
T1	1 (5.3)	14 (73.7)	3 (15.7)	1 (5.3)	0.532^c^	0.166^f^
T2	1 (5.3)	9 (47.35)	9 (47.35)	0 (0)		

*Note:* KRAS IHC^+^, KRAS protein present; KRAS IHC^**−**^, KRAS protein absent; N_sup_, superficial layer of adjacent normal tissue; N_cry_, base of Lieberkühn crypts in adjacent normal tissue; T1, tumour center; T2, invasive tumour front; letter marks the difference between.

^a^ N_sup_ and N_cry_.

^b^ N_sup_ and T1.

^c^ N_sup_ and T2.

^d^ N_cry_ and T1.

^e^ N_cry_ and T2.

^f^ T1 and T2. *p* values were obtained by Fisher's exact test (*p < 0.05, **p < 0.01, ***p < 0.001, ****p < 0.0001).

**FIGURE 3:**
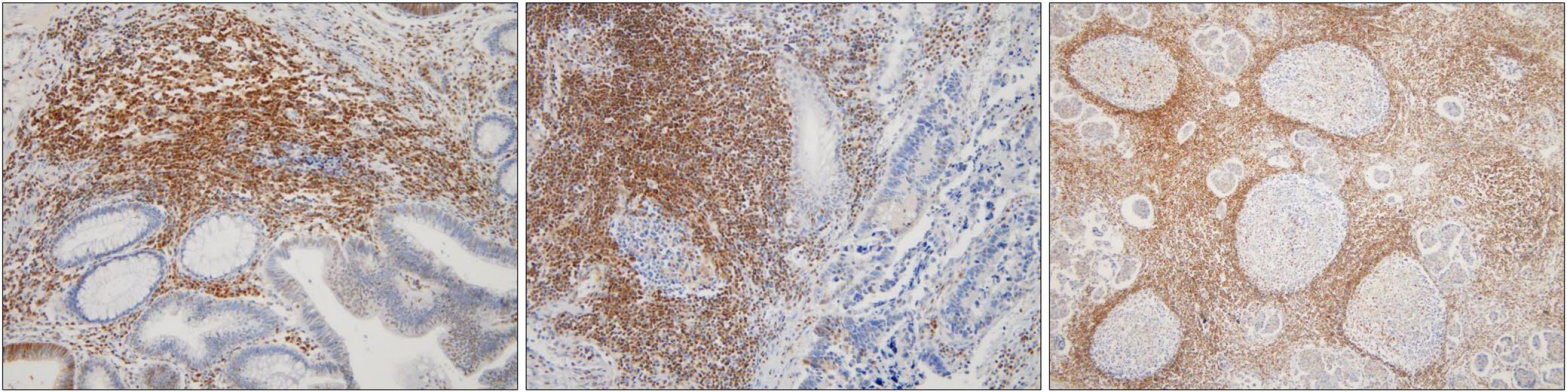
Immunohistochemical expression of p27 in colorectal cancer samples. Infiltration of immune cells positive for p27 protein expression into the tumour microenvironment (TME) (magnification, ×200).

Interestingly, we observed a substantial infiltration of immune cells expressing p27 in tumour samples (Figure 3). This finding could explain the discrepancy between gene expression data, which indicated elevated p27 expression in tumour tissue compared to normal tissue and immunohistochemical analyses, which did not show a corresponding increase in p27 protein levels within tumour cells.

To investigate the presence of p27 in WT KRAS CRC we assessed its subcellular localization and examined its association with KRAS protein expression. Our IHC analysis showed that p27 was localized mainly in the nucleus; however, a subset of samples displayed cytoplasmic staining in addition to nuclear staining. In the basal regions of the Lieberkühn crypts within adjacent normal tissue, p27 exhibited a relatively equal distribution between the nucleus and cytoplasm. In contrast, in the superficial epithelial layer of the same tissue, p27 was predominantly localized in the nucleus, irrespective of KRAS protein expression status. In samples without KRAS protein presence (IHC^−^), the proportion of cytoplasmic p27‐positive cells in T1 and T2 samples was comparable to that in normal tissues. In contrast, samples with detectable KRAS protein expression (IHC^+^) exhibited a significantly higher cytoplasmic p27 expression in T2 samples when compared to the superficial epithelial layer of adjacent normal tissue (*p* = 0.001) and tumour center tissue (*p* = 0.037; Table 2).

**TABLE 2:** P27 IHC localization in normal (N_sup_ and N_cry_), tumour center and invasive tumour front tissues stratified by WT KRAS protein expression.

27 location	N	C	*p*	
KRAS IHC+				
Nsup	25 (80.6)	6 (19.4)	0.091^a^	0.585^d^
Ncry	17 (54.8)	12 (45.2)	0.376^b^	0.196^e^
T1	20 (64.5)	9 (35.5)	**0.001** ^c^**	**0.037** ^f^*
T2	12 (38.7)	18 (61.3)		
KRAS IHC^**−**^				
N_sup_	14 (73.7)	5 (26.3)	0.313^a^	0.078^d^
N_cry_	10 (52.6)	9 (47.4)	0.693^b^	0.507^e^
T1	15 (78.9)	3 (21.1)	0.728^c^	0.443^f^
T2	12 (63.2)	6 (36.8)		

*Note:* N, nuclear staining, C, cytoplasmic staining; KRAS IHC+, KRAS protein present; KRAS IHC‐, KRAS protein absent; Nsup, superficial layer of adjacent normal tissue; Ncry, base of Lieberkühn crypts in adjacent normal tissue; T1 tumour center; T2 invasive tumour front; letter marks the difference between.

^a^ N_sup_ and N_cry_.

^b^ N_sup_ and T1.

^c^ N_sup_ and T2.

^d^ N_cry_ and T1.

^e^ N_cry_ and T2.

^f^ T1 and T2. *p* values were obtained by Fisher`s exact test (*p < 0.05, **p < 0.01).

These findings indicate that in WT KRAS CRC, both the regional distribution and subcellular localization of p27 correlate with KRAS protein expression. Therefore, we sought to investigate whether p27 expression is associated with clinicopathological features of WT KRAS CRC.

### 3.3 Cytoplasmic p27 Expression Has no Effect on Clinicopathological Features in Patients With WT KRAS CRC

Table 3 presents the association between cytoplasmic p27 expression in CRC tissues and various clinicopathological parameters. No significant association was observed between cytoplasmic p27 expression and patient age, gender, tumour size, histological grade, or Dukes' stage. These findings suggest that cytoplasmic localization of p27 in WT KRAS CRC does not appear to be associated with specific clinicopathological characteristics.

**TABLE 3:** Clinicopathological characteristics of 50 patients with sporadic WT KRAS colorectal cancer, stratified by the presence or absence of cytoplasmic p27 protein expression in the tumour center (T1).

Characteristic	Cytoplasmic p27		*p*
	Negative	Positive	
Age			
< 65 years	23 (74.2)	8 (25.8)	> 0.999
≥ 65 years	15 (78.9)	4 (21.1)	
Gender			
Male	23 (79.3)	6 (20.7)	0.738
Female	15 (71.4)	6 (28.6)	
Tumour size			
≤ 5 cm	22 (73.3)	8 (26.7)	0.740
> 5 cm	16 (80.0)	4 (20.0)	
Histological grade			
Well (1)	15 (75.0)	5 (25.0)	0.605
Moderate (2)	20 (74.0)	7 (26.0)	
Poor (3)	3 (100.0)	0 (0.0)	
Dukes' stage			
A	4 (100.0)	0 (0.0)	0.403
B	10 (66.7)	5 (33.3)	
C	16 (72.7)	6 (27.3)	
D	8 (88.9)	1 (11.1)	
Tumour location			
Left	35 (74.4)	12 (25.6)	0.562
Right	3 (100.0)	0 (0.0)	

*Note:* *p* values were obtained by Fisher's exact test.

### 3.4 p27 Expression Does Not Correlate With *Hsa‐miR‐221‐3p* and *Hsa‐miR‐222‐3p* Expression

Given that *miR‐221‐3p* and *miR‐222‐3p* are frequently upregulated in tumours and target *CDKN1B* mRNA, we assessed their expression levels in 50 wild‐type KRAS colorectal cancer (CRC) samples and their matched adjacent normal tissues. Additionally, we investigated the correlation between the expression of these microRNAs and p27 protein levels.

**FIGURE 4:**
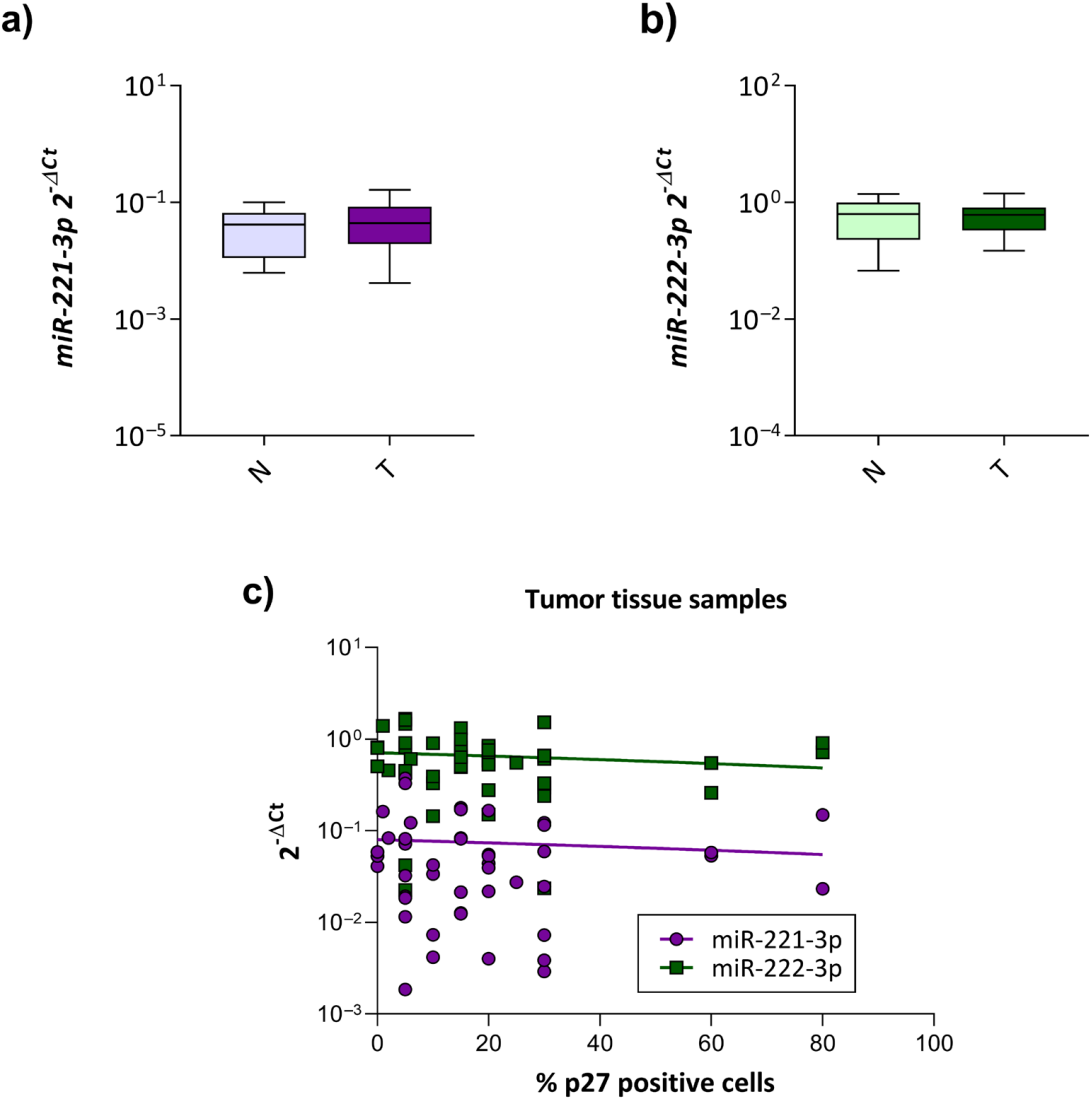
miR‐221‐3p and miR‐222‐3p expression in normal and tumour tissue. Box plots show the expression of (a) miR‐221‐3p and (b) miR‐222‐3p in adjacent normal tissue (N) and tumour center (T) of WT KRAS CRC. (c) The correlation between the percentage of *p27*‐positive cells and expression of miR‐221‐3p and miR‐222‐3p was analysed in tumour tissues. (a, b) Data are presented as box‐and‐whisker plots (5th–95th percentiles) and were analysed using Wilcoxon signed‐rank test. (c) Spearman's rank correlation and linear regression were applied.

No statistically significant differences were observed between tumour and adjacent normal tissues in *miR‐221‐3p* (*p* = 0.204, Figure 4a) and *miR‐222‐3p* (*p* = 0.518, Figure 4b) expression. Furthermore, no significant correlation was found between the percentage of p27 positive cells and either *miR‐221‐3p* or *miR‐222‐3p* in tumour tissues (*p* = 0.805 and *p* = 0.2172, respectively; Figure 4c).

### 3.5 *CDKN1B* Gene Polymorphism *V109G* Shows no Association With Risk of Colorectal Cancer

We analysed the relationship between *CDKN1B V109G* (rs2066827) polymorphism located in exon 2 and the risk of WT KRAS colorectal cancer susceptibility.

HWE analysis showed no significant deviation in the healthy control group (χ^2^ = 1.268, *p* = 0.260), confirming the reliability of genotype data and suitability of the control population for association analysis. Logistic regression analysis revealed no significant relationships with the *V109G* variant and CRC risk in our study population (Table 4).

**TABLE 4:** Genotype frequencies of *CDKN1B* (*109VG*) polymorphism in WT KRAS CRC patients and healthy controls.

*p27 T326G (V109G)*	Controls 180 (%)	WT KRAS CRC 150 (%)	OR (95% CI)	*p*
TT	110 (61.1)	89 (59.3)	1	0.620
TG	58 (32.2)	54 (36.0)	1.151 (0.7333–1.804)	
GG	12 (6.7)	7 (4.7)	0.721 (0.2810–1.846)	
TT	110 (61.1)	89 (59.3)	1	0.821
TG + GG	70 (38.9)	61 (40.7)	1.055 (0.6967–1.661)	
T	278 (77.2)	232 (77.3)	1	> 0.9999
G	82 (22.8)	68 (22.7)	1.140 (0.6855–1.429)	

*Note:* *p* values were obtained by Fisher's exact test. OR, odds ratio; CI, confidence interval.

Abbreviations: CI, confidence interval; OR, odds ratio.

## 4 Discussion

The cell cycle is regulated through the sequential activation and assembly of multiple regulatory protein complexes. The Cip/Kip family of CDKIs broadly inhibits various cyclin‐CDK complexes thereby inducing cell cycle arrest and ultimately preventing cellular proliferation. Among these, p27 is a key CDKI whose expression is markedly reduced in various malignancies [7]. Several studies showed that in various tumour types, including colon cancer, p27 undergoes translocation from the nucleus, resulting in elevated cytoplasmic accumulation [33, 34]. In light of these findings, our study investigated expression and subcellular localization of p27 in sporadic WT KRAS CRC, with additional evaluation of its relationship to KRAS protein status, clinicopathological parameters as well as p27 regulation by *miR‐221‐3p, miR‐222‐3p* and *CDKN1B* polymorphism *V109G*.

In the present study, we observed that *CDKN1B* expression in both tumour and adjacent normal tissues was similar in KRAS‐mutated samples. In contrast, in wild‐type KRAS samples, there was a slight increase in *CDKN1B* expression in tumour tissue compared to adjacent normal tissue; however, this difference did not reach statistical significance. At the protein level, in WT KRAS CRC we observed a strong contrast in expression patterns, with an inverse correlation between the gene and protein expression profiles. However, previous studies also indicate that p27 protein expression does not always correlate with gene expression levels, both in cell lines [35] and tumour samples [8, 36]. Similarly, prior studies have demonstrated that p27 expression does not correlate with MSI status or specific oncogenic alterations, but rather shows broad variability across CRC cases [6]. We hypothesize that this gene–protein discrepancy may be attributed to the observed high influx of strongly p27‐positive immune cells in tumour samples. Importantly, this should not be interpreted as an indication of cell cycle arrest, as p27 is a known regulator of lymphocyte proliferation and immune responses [37]. The prominent presence of p27‐positive immune infiltrates at the invasive front may therefore represent a biologically relevant feature of local immune surveillance in WT KRAS tumours, although its relationship with immune subtypes or clinical outcomes remains beyond the scope of the present study.

Our results showed that the highest percentage of p27 positive cells in WT KRAS CRC was present in the superficial layer of adjacent normal mucosa, while it was reduced in tumour cells, consistent with previous studies suggesting a progressive decline of p27 in tumorigenesis [8, 38]. Consistent with previously published studies on healthy colonic tissue [39, 40] we also observed that p27 expression in adjacent normal mucosa was predominantly localized to superficially differentiated epithelial cells and gradually decreased along the crypt axis. In contrast, within the crypt epithelium, which harbours a heterogeneous cell population, including stem cells at the crypt base, only a small number of p27‐positive cells were detected. However, these cells exhibited the highest staining intensity, supporting the proposed role of p27 in intestinal epithelial differentiation [41, 42]. Although the relatively limited cohort size restricts the strength of the conclusions, post hoc power analysis confirmed adequate power to detect the major differences in p27 expression between normal and tumour compartments, which represent the primary findings of this study. However, smaller differences between anatomically adjacent compartments, particularly between the tumour center and invasive front and between cryptal epithelium and tumour regions, were underpowered, especially within the IHC^−^ subgroup, indicating that larger cohorts will be required for definitive validation.

In general, the association between KRAS and p27 is complex and context‐dependent, particularly in colorectal cancer. In KRAS/BRAF‐mutated CRC subtypes, loss of p27 is more common and it was shown that it can influence a patient's response to CDK4/6 inhibitors [43, 44]. In contrast, the role of WT KRAS in the regulation of p27 remains poorly understood. Several studies have examined p27 expression in CRC cell lines, encompassing both WT KRAS (HT29, Caco‐2, Colo205, Colo320) and mutated KRAS (SW480, DLD1, HCT116, RKO, LoVo, HCT15, T84) cell lines. However, results revealed that the levels of p27 expression were highly variable across cell lines, irrespective of KRAS or BRAF mutation status [6, 35, 45]. In this context, our analysis provides new insights into this relationship, showing that tumours with detectable WT KRAS protein have increased p27 presence at the invasive tumour front, suggesting a potential influence of KRAS signalling on regional p27 distribution.

In contrast to its nuclear form, cytoplasmic p27 has been implicated in promoting tumourigenesis in several human cancers through mechanisms that do not involve the inhibition of cyclin‐dependent kinases [16, 46, 47]. KRAS mutations do not directly influence the nuclear localization of p27. Instead, activation of the PI3K/AKT pathway, which is downstream of KRAS, can phosphorylate p27, promoting its retention in the cytoplasm and thereby inhibiting its tumour‐suppressive function in the nucleus. Our study demonstrated that cytoplasmic p27 was more present at the invasive front of tumours with detectable KRAS protein expression, whereas tumours without KRAS protein presence showed nuclear‐dominant p27. However, due to the limited subgroup sizes, the study was not sufficiently powered for robust between‐group comparisons; therefore, no definitive conclusions regarding differences between KRAS IHC^+^ and KRAS IHC^−^ tumours can be drawn. While the present study did not include functional experiments to directly assess the consequences of p27 cytoplasmic relocalization, it is tempting to speculate that this spatial redistribution, particularly at the invasive tumour front, may reflect a contextual shift in p27 function, from nuclear cell cycle regulation towards cytoplasmic roles previously implicated in the promotion of cell motility and invasiveness.

Despite the above‐mentioned patterns, cytoplasmic p27 expression did not significantly correlate with age, gender, tumour size, grade, or Dukes' stage, suggesting that its altered localization is not linked to clinicopathological features in WT KRAS CRC. Nevertheless, the limitation of this study is the absence of survival data, as the follow‐up period was insufficient to enable reliable analysis of overall or disease‐free survival and to assess the prognostic relevance of the observed cytoplasmic p27 localization patterns. To date, studies have primarily investigated the correlation between p27 expression and clinicopathological features, rather than examining p27 localization as an independent variable. In a recent meta‐analysis study investigating the relationship between p27 expression and prognostic factors in colon cancer, Zou et al. reported that right colon tumours had lower p27 expression. Furthermore, patients with high p27 expression, which is often indicative of intact cell cycle control mechanisms, exhibited significantly improved overall survival compared to those with low expression. However, they showed that p27 expression had no effect on TNM staging, lymph node metastasis, or tumour size [34]. Similarly, Hershko and Shapira showed that, in general, most studies agree that high levels of p27 expression are associated with a good overall survival, whereas low levels are associated with aggressive tumour behaviour and poor clinical outcome [48]. Despite the potential relevance of p27 subcellular localization, only two studies have investigated its cytoplasmic expression in relation to clinical outcomes, and they showed that it is associated with the presence of MSI/CIMP [44] and overall better prognosis [49]. In our study, all samples were confirmed microsatellite stable (MSS), eliminating MSI status as a confounding factor. Although CIMP status was not directly assessed, the exclusion of MSI‐positive and MLH1‐methylated tumours substantially reduces the likelihood of CIMP‐high confounding, given the strong co‐occurrence of these phenotypes. Our findings, therefore, suggest that altered cytoplasmic p27 localization in WT KRAS CRC may occur independently of the MSI/CIMP molecular background previously linked to this expression pattern.

Previous studies have demonstrated that elevated levels of *miR‐221* and *miR‐222* are linked to reduced expression of the tumour suppressor protein p27, thereby promoting cell proliferation and facilitating cancer progression in a variety of malignancies [23, 50, 51, 52]. In colon cancer cell lines, high expression of *miR‐221* and *miR‐222* was also shown to promote tumour growth by directly targeting the tumour suppressor p27, leading to its downregulation. This suppression of p27 reduced the inhibition of the cell cycle, allowing for increased cell proliferation and colony formation [53]. Given the known post‐transcriptional regulation of p27 by *miR‐221‐3p* and *miR‐222‐3p*, we assessed their expression and potential regulatory role in WT KRAS CRC. While *CDKN1B* was identified as a high‐confidence target of both miRNAs *in silico*, our analysis revealed no significant differences in *miR‐221/222* expression between tumour and normal tissue, nor any correlation with p27 protein levels. The lack of correlation between *miR‐221/222* expression and p27 levels in our WT KRAS cohort contrasts with findings in KRAS‐mutated CRC, where activated RAS–ERK and JAK‐STAT3 signalling pathways promote *miR‐221/222* upregulation and *CDKN1B* suppression [53]. This suggests that the *miR‐221/222*–p27 regulatory axis may be specific to activated RAS signalling, while alternative mechanisms, including post‐translational regulation and cytoplasmic sequestration, may predominate in WT KRAS tumours. These findings further support molecular subtype‐specific regulation of p27 in CRC.

The *CDKN1B V109G* (rs2066827) polymorphism is a single nucleotide variant in the *CDKN1B* gene, resulting in a substitution of valine for glycine at codon 109. This polymorphism has been investigated for its potential association with various malignancies [54]; however, the findings remain inconclusive and its correlation with an elevated risk of CRC or potential less favourable survival outcomes is not consistently supported in the literature [55, 56]. We also have not observed any association with *CDKN1B V109G* (rs2066827) SNP and CRC susceptibility in our cohort.

While the relatively limited sample size of our patient cohort restricts the strength of the conclusions that can be drawn, our data suggest that p27 protein expression and its subcellular distribution are influenced by KRAS protein status even in the absence of KRAS mutations, pointing towards a potential role for WT KRAS signalling in modulating p27 localization.

## Author Contributions

**Iva Paladin:** investigation, formal analysis, writing – original draft. **Sonja Marinović:** investigation, formal analysis, writing – original draft, visualization. **Tina Catela Ivković:** formal analysis, investigation, methodology. **Anita Škrtić:** data curation, resources, formal analysis. **Sanja Kapitanović:** writing – review and editing, conceptualization, funding acquisition. **Donatella Verbanac:** writing – review and editing, supervision.

## Funding

The present study was supported by the Hrvatska zaklada za znanost (Croatian Science Foundation) (grant number HRZZ‐IP‐2016‐06‐1430).

## Ethics Statement

The study was approved by the Ethical Committees of Clinical Hospital Merkur, Zagreb, the Medical School, and the Faculty of Pharmacy and Biochemistry at the University of Zagreb, and was conducted in accordance with the Helsinki Declaration.

## Consent

Written informed consent was obtained from all patients included in the study.

## Conflicts of Interest

The authors declare no conflicts of interest.

## Data Availability

The data that support the findings of this study are available from the corresponding author upon reasonable request.
